# Redox-stat bioreactors for elucidating mobilisation mechanisms of trace elements: an example of As-contaminated mining soils

**DOI:** 10.1007/s00253-018-9165-4

**Published:** 2018-06-21

**Authors:** Liwia Rajpert, Andreas Schäffer, Markus Lenz

**Affiliations:** 1 Institute for Ecopreneurship, School of Life Sciences, University of Applied Sciences and Arts Northwestern Switzerland, Gründenstrasse 40, 4132 Muttenz, Switzerland; 2 Institute for Environmental Research (Biology V), RWTH Aachen University, 52074 Aachen, Germany; 3 Sub-Department of Environmental Technology, Wageningen University, 6700 EV Wageningen, The Netherlands

**Keywords:** Arsenic remediation, Redox-stat bioreactor, Trace element fate

## Abstract

The environmental fate of major (e.g. C, N, S, Fe and Mn) and trace (e.g. As, Cr, Sb, Se and U) elements is governed by microbially catalysed reduction-oxidation (redox) reactions. Mesocosms are routinely used to elucidate trace metal fate on the basis of correlations between biogeochemical proxies such as dissolved element concentrations, trace element speciation and dissolved organic matter. However, several redox processes may proceed simultaneously in natural soils and sediments (particularly, reductive Mn and Fe dissolution and metal/metalloid reduction), having a contrasting effect on element mobility. Here, a novel redox-stat (*R*_cont_) bioreactor allowed precise control of the redox potential (159 ± 11 mV, ~ 2 months), suppressing redox reactions thermodynamically favoured at lower redox potential (i.e. reductive mobilisation of Fe and As). For a historically contaminated mining soil, As release could be attributed to desorption of arsenite [As(III)] and Mn reductive dissolution. By contrast, the control bioreactor (*R*_nat_, with naturally developing redox potential) showed almost double As release (337 vs. 181 μg g^−1^) due to reductive dissolution of Fe (1363 μg g^−1^ Fe^2+^ released; no Fe^2+^ detected in *R*_cont_) and microbial arsenate [As(V)] reduction (189 μg g^−1^ released vs. 46 μg g^−1^ As(III) in *R*_cont_). A redox-stat bioreactor thus represents a versatile tool to study processes underlying mobilisation and sequestration of other trace elements as well.

**Electronic supplementary material:**

The online version of this article (10.1007/s00253-018-9165-4) contains supplementary material, which is available to authorized users.

## Introduction

In the former mining area of the Złoty Stok (Lower Silesia, southwest Poland), long-lasting mining activities together with lack of reclamation resulted in As contamination (> 2000 μg g^−1^ soil) of the nearby valley (i.e. Trująca Valley) (Krysiak and Karczewska 2007). Elevated concentrations of As, Hg, Cr and/or Mn were found in surface and ground waters (Marszałek and Wąsik 2000). Nevertheless, the lower part of the Trująca Valley is still used agriculturally, which may pose a risk of As entry into the human food chain (Krysiak and Karczewska 2007; Karczewska et al. 2013). Potential mobility and bioavailability of As are dependent on As speciation and the physicochemical properties of the host matrix (i.e. identity and quantity of sorbing phases) (Borch et al. 2010). Under oxidising conditions, which prevail in top soils during dry conditions, As is thermodynamically favoured to exist in the form of negatively charged arsenate [As(V)], which has a high affinity to Fe/Mn-(oxyhydr)oxides (Ehlert et al. 2014; Couture et al. 2015). Upon water logging (e.g. as a result of flooding or rainfall after soil compaction), reducing conditions can be induced by decreased diffusion and fast microbial consumption of residual atmospheric oxygen. Such reducing conditions favour microbially catalysed reduction of arsenate to arsenite [As(III)] (by dissimilatory arsenate reducing bacteria), which can result in As mobilisation owing to the low sorption capacity on major soil phases (Giménez et al. 2007). Further, Fe/Mn-(oxyhydr)oxides are prone to microbially catalysed reductive dissolution therewith mobilising sequestered As (as well as other trace elements and radionuclides) (Ehlert et al. 2014; Couture et al. 2015; Zhang et al. 2014).

Mesocosms are typical laboratory systems used to study mechanisms of As and other trace element mobilisation for risk assessment. Typically, contaminated soil/sediment slurry samples are incubated over time, and conclusions on the underlying mobilisation and sequestration processes are made on the basis of correlations between biogeochemical proxies (e.g. dissolved Fe and Mn, dissolved organic matter, pH, redox potential) and the elements mobilised (as either the total element mobilised or elemental species) (Masscheleyn et al. 1991; Kneebone and Hering 2000; Hockmann et al. 2014). In biogeochemistry, the redox potential (*E*_h_) is measured as it reflects the tendency of a system to undergo redox reactions and provides support for conclusions by thermodynamic equilibrium modelling of favoured species. If not supported by further analysis, such as spectroscopy and molecular biology analysis, the conclusions obtained may be biased because several (redox) reactions may overlay (particularly in structurally heterogeneous systems) or be kinetically hindered, although thermodynamically favoured. However, *E*_h_ not only serves as a proxy of prevalent reactions that occur in a system indicating the availability of electrons in the system, but can also be used to actively suppress unwanted reactions by making the reaction thermodynamically unfavoured. More precisely, soil/sediment slurries that are not in contact with ambient oxygen tend to a decreasing *E*_h_ because of the consumption of organic electron donors (either natural or supplied). Then by feeding finite amounts of a preferred external electron acceptor (oxygen), the *E*_h_ can be ultimately maintained at intermediary values between fully reducing and fully oxidising conditions. This renders all redox reactions occurring at a lower potential thermodynamically unfavourable.

In this study, such a redox-stat bioreactor was designed to quantify the contributions of Fe,Mn-(oxyhydr)oxide reductive dissolution and arsenate reduction on overall As mobilisation in historically contaminated mining soils. In the redox stat (*R*_cont_), E_h_ was controlled at a true steady state where in-between fully oxidising and fully reducing conditions (~ +150 mV) are maintained, allowing only for Mn reduction while supressing Fe and arsenate reduction. Elemental mobilisation as well as speciation in the developed reactor was compared with that of a control reactor with naturally developing redox potential (*R*_nat_) by inductively coupled plasma mass spectrometry (ICP-MS), X-ray fluorescence (XRF) and liquid chromatography-ICP-MS (LC-ICP-MS).

## Materials and methods

### Source of soil, sampling and calculation of elemental mobilisation

Soil samples were collected from a surface layer (0–20 cm) on the bank of the Trujaca River (“Poisonous Stream”, drain of the former goldmine) and sample preparation was performed as described previously (Rajpert et al. 2016). Total element concentrations (using XRF), element mobilisation rates (μg L^−1^ h^−1^) and cumulative element mobilised (μg g^−1^ of soil) were determined as described previously (Rajpert et al. 2016).

### Bioreactor operation

Two continuous stirred-tank reactors (CSTR, Multifors, Infors HT, Bottmingen, Switzerland) were operated at a slurry concentration of 10% soil (*w*/*v*) under mesophilic (21 ± 5 °C) and slightly alkaline conditions (pH = 8.0 ± 0.6, using automated acid or base dosing) for 1368 h (57 days). The hydraulic retention time was set to 48 h. The reactors were continuously fed with filtered (0.22 μm), degassed minimal medium (Rajpert et al. 2016). Lactate (0.11 g L^−1^) was added at an organic loading rate of 48 mg COD L^−1^ day^−1^ (Chemical Oxygen Demand), supplying an excess of electrons based on the total As concentration. A constant flow of N_2_ was maintained through the reactor headspace during operation. In both reactors, the redox potential was monitored with a silver-free gel electrode (QIS, Oosterhout, The Netherlands). The redox potential in the first reactor developed naturally over time (referred to as “*R*_nat_”), while in the second reactor, the redox potential was controlled (referred to as “*R*_cont_”). The set-point of + 160 mV was maintained by short doses of synthetic air N_2_:O_2_ (80:20) at 3 L min^−1^. Air flow was switched on/off by a controller (Standard Digital Redox—Processor No: 2/VIII; Trop-Electronic GmbH, Luedenscheid, Germany) via a 240-V solenoid valve.

### Liquid phase analysis

Samples (~ 20 mL) were sequentially centrifuged (4500 rcf, 10 min, 21 °C) and filtered (sequential; 0.45 and 0.2 μm pore size, Whatman, Hertogenbosch, The Netherlands). The concentration of total dissolved As, Mn and Fe were quantified on an Agilent 7500cx ICP-MS (Agilent Technologies AG, Basel, Switzerland) with standard settings (Zimmermann et al. 2013) at masses ^56^Fe, ^55^Mn and ^75^As. Mn and As were measured in the collision mode using helium, whereas Fe was measured in the reaction mode using H_2_. Speciation analysis was performed by LC-ICP-MS, as previously described, after preservation of the samples in EDTA/AcOH (Gallagher et al. 2004). Preserved samples were stored at 4 °C and measured within 72 h after sampling. The concentration of dissolved iron (Fe^2+^) was measured spectrophotometrically with 1.10-phenantroline after preservation in 0.5 M HCl (Fadrus and Malý 1975).

## Results

### Element mobilisation

**Fig. 1:**
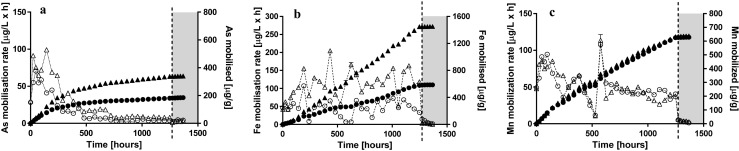
Mobilisation of As (**a**), Fe (**b**) and Mn (**c**) in *R*_cont_ (circles) and *R*_nat_ (triangles) in terms of elemental mobilisation rate (open symbols, primary *Y*-axis) and total element mobilised (solid symbols, secondary *Y*-axis). The dashed line is representing the beginning of oxidising conditions

**Fig. 2:**
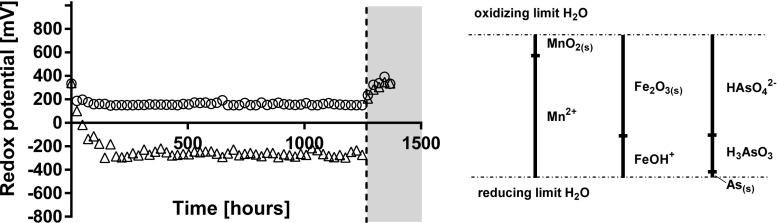
Redox potential in *R*_cont_ (circles) and *R*_nat_ (triangles). The dashed line is showing the beginning of oxidising conditions. The solid lines (right side) are representing the thermodynamically favoured species at pH 8.0 for Mn, Fe and As (FACT/FACTSAGE; ∑As/Fe/Mn = 10^−10^, 298.15 K, 10^5^ Pa; Mn/Fe or As-O-H system, resp.) (Takeno 2005)

The mobilisation of As in *R*_nat_ showed initially high rates (maximal mobilisation rate 99.1 μg L^−1^ h^−1^ at 144 h) followed by a gradual decrease and a nearly constant, low As release until the end of reactor operation (minimal mobilisation rate 1.4 μg L^−1^ h^−1^ at 1272 h) (Fig. 1a). By contrast, both Fe and Mn were released steadily throughout the reactor operation (indicated by the straight lines in cumulative element released, Fig. 1b, c). Fe reducing conditions developed during the first ~ 192 h (Fig. 2), resulting in steadily increasing Fe mobilisation; whereas later, Fe was mobilised rather constantly (average 119.0 ± 35.8 μg L^−1^ h^−1^, maximal mobilisation rate 204.5 μg L^−1^ h^−1^ at 432 h) until the introduction of oxidising conditions (+ 0.2 ± 0.3 V) at 1272 h (Fig. 1b). Mn reducing conditions developed more rapidly (from the start of the reactor operation), resulting initially in high mobilisation rates (maximal mobilisation rate 85.3 μg L^−1^ h^−1^ at 48 h), followed by a gradual decrease. An exceptionally high Mn mobilisation rate (113 μg L^−1^ h^−1^) was observed on a single sampling only and was potentially an outlier, since it occurred in both reactors. For the remaining reactor operation, Mn mobilisation was rather constant at around 40 μg L^−1^ h^−1^ (Fig. 1c). Overall, 337 μg g^−1^ of As (~ 17% of total As), 1448 μg g^−1^ of Fe (~ 3% of total Fe) and 632 μg g^−1^ of Mn (~ 54% of total Mn) were mobilised during the *R*_nat_ reactor operation until re-oxidation (Fig. 1a–c and Table 1).

**Table 1:** Cumulative element mobilisation [μg/g] after 1248 h (52 days) of reactor operation

	As total dissolved*	As(III)*	As(V)*	Fe total dissolved	Fe colloid**	Fe^2+^ dissolved	Mn total dissolved
*R*_nat_	337	189	144	1448	85	1364	632
*R*_cont_	181	46	132	578	578	0	625

*As total dissolved by ICP-MS, As(III)/As(V) by LC-ICP-MS

**Fe colloid = Fe total − Fe^2+^

In *R*_cont_, initial As mobilisation was lower and decreased faster in comparison to *R*_nat_ (Fig. 1a). Fast mobilisation (comparable to *R*_nat_) was observed only until ~ 96 h, illustrated by the virtually identical lines of cumulative element mobilised (Fig. 1a). Subsequently, As mobilisation rate decreased until ~ 500 h, after which hardly any As was mobilised (minimal mobilisation rate 2.3 μg L^−1^ h^−1^ at 1272 h; Fig. 1a). As mobilisation rates in *R*_cont_ remained lower than in *R*_nat_ throughout the reactor operation. In contrast to *R*_nat_, the Fe mobilisation rate in *R*_cont_ was lower and relatively stable during the reactor operation with an average mobilisation of 46.6 ± 21.5 μg L^−1^ h^−1^ (maximal mobilisation rate 107.2 μg L^−1^ h^−1^ at 192 h, minimal mobilisation rate 7.2 μg L^−1^ h^−1^ at 576–624 h; Fig. 1b). Dissolution of manganese in the *R*_cont_ reactor was quite similar to the *R*_nat_ (Fig. 1c). Overall, 181 μg g^−1^ of As (~ 9% of total As), 578 μg g^−1^ of Fe (~ 1% of total Fe, exclusively as colloidal Fe) and 625 μg g^−1^ of Mn (~ 54% of total Mn) were mobilised during the *R*_cont_ reactor operation (Fig. 1a–c and Table 1).

Upon re-oxidation, both reactors showed similar effects on Fe and Mn mobilisation rates, decreasing to virtually zero (Fig. 1b, c), whereas there was already hardly any As mobilised before oxidation (Fig. 1a).

### Redox potential and speciation analysis

**Fig. 3:**
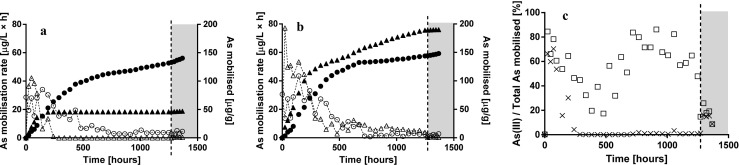
Arsenic speciation in *R*_cont_ (**a**) and *R*_nat_ (**b**). As(V) (circles; mobilisation rate in open; cumulative mobilised in solid) and As(III) (triangles; mobilisation rate in open; cumulative mobilised in solid). The dashed line is showing the beginning of oxidising conditions. Share of As(III) on total arsenic mobilised [%] (*R*_nat_ in squares, *R*_cont_ in crosses) (**c**)

*R*_cont_ indeed allowed to precisely control *E*_h_ at 159 ± 11 mV over a period of > 50 days, whereas in *R*_nat_, the redox potential decreased due to microbial activity to As(V)-reducing and Fe(III)-reducing (both ~ − 120 mV) conditions from around 72 h (Fig. 2). At the conditions specified in Takeno (2005), reduced species (H_3_AsO_3_; arsenite; As(III) and FeOH^+^; Fe^2+^) were favoured thermodynamically in *R*_nat_ already after 72 h of operation. Indeed, there was a sustained release of As(V) in *R*_cont_ during the entire reactor operation (Fig. 3a), whereas hardly any As(V) released in *R*_nat_ after 720 h until re-oxidation. The apparent difference in As speciation becomes even more visible when considering the share of As(III) in the total As released in the reactors (Fig. 3c).

The dissolution of both As(III) and As(V) in *R*_nat_ was characterised by high mobilisation rates initially (0–240 h) [As(III) maximal mobilisation rate 77.2 μg L^−1^ h^−1^ at 24 h, As(V) maximal mobilisation rate 43.4 μg L^−1^ h^−1^ at 144 h; Fig. 3b]. At 288 h, concentrations of both species in the reactor effluent started to decrease steadily and this trend continued until the end of the reactor operation (Fig. 3b; As(III) minimal mobilisation rate 2.8 μg L^−1^ h^−1^ at 480 h, As(V) minimal mobilisation rate 0.7 μg L^−1^ h^−1^ at 768 h). Overall, 148.2 μg g^−1^ of As(V) (7% of total As) and 190.0 μg/g of As(III) (9% of total As) were mobilised in the *R*_nat_ reactor. In *R*_cont_ also there were high mobilisation rates of both As(V) and As(III) initially [As(V) maximal mobilisation rate 42.3 μg L^−1^ h^−1^ at 48 h, As(III) maximal mobilisation rate 34.1 μg L^−1^ h^−1^ at 144 h]. The mobilisation rate of the As(III) dropped drastically starting from 240 h onwards [As(III) was not detected within 336–672 h; Fig. 3a]. Overall, 140.5 μg g^−1^ of As(V) (7% of total As) and 47 μg g^−1^ of As(III) (2% of total As) were mobilised in *R*_cont_ (Fig. 3a).

**Fig. 4:**
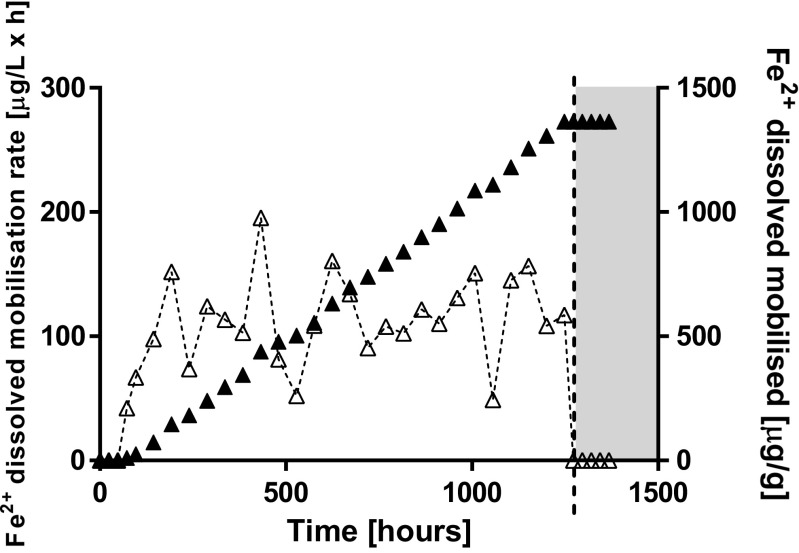
Fe^2+^ in *R*_nat_ in terms of elemental mobilisation rate (open symbols, primary *Y*-axis) and total element mobilised (solid symbols, secondary *Y*-axis). The dashed line is representing the beginning of oxidising conditions. Note, that no Fe^2+^ was detected in *R*_cont_

Fe speciation was clearly different in *R*_nat_ and *R*_cont_. Despite the total Fe mobilised (Fig. 1b) in *R*_cont_, reduced Fe^2+^ was found exclusively in *R*_nat_ (Fig. 4). Reduced Fe^2+^ was released starting at 72 h and throughout the reactor operation with an average rate of 93 ± 54 μg L^−1^ h^−1^ (maximal mobilisation rate 195.4 μg L^−1^ h^−1^ at 432 h, minimal mobilisation rate 48.9 μg L^−1^ h^−1^ at 1056 h; Fig. 4). Overall, 1363.8 μg g^−1^ of reduced Fe (2.5% of the total Fe) were mobilised in the *R*_nat_ reactor (Fig. 4).

### Experimental elemental recovery

An XRF analysis showed considerable quantities of As (2010 μg g^−1^), Fe (53,867 μg g^−1^) and Mn (1164 μg g^−1^) in the initial soil (SI, Table S1). The soil residue after *R*_nat_ reactor operation contained 1625 μg g^−1^ of As, 548 μg g^−1^ of Mn and 49,437 μg g^−1^ of Fe, whereas *R*_cont_ reactor showed higher residual As and Fe concentrations (1727.0 μg g^−1^ and 51,427 μg g^−1^, respectively), and comparable Mn content (606 μg g^−1^) (SI, Table S1). The differences in element concentrations between the initial and treated soil corresponded well to the cumulative element mobilised measured in the *R*_nat_ and *R*_cont_ reactor effluents (Table 1), resulting in closed element balances (experimental recoveries 95–106%, Table S1). The experimental recovery for As species (i.e. As(III) and As(V) in comparison to total dissolved As) was generally around 100% (SI, Fig. S1).

## Discussion

In this work, we applied for the first time a true redox-stat reactor to study mechanisms underlying trace element release of As from contaminated mining soils. The redox potential was constant (159 ± 11 mV, Fig. 2) for approximately 2 months of operation. The relatively high redox potential chosen in *R*_cont_ favoured Mn reductive dissolution only, whereas both As(V) reduction and Fe reductive dissolution should not be thermodynamically favoured (Fig. 1c). Indeed, Mn mobilisation was virtually the same in both reactors (*R*_cont_ = 624 μg g^−1^ vs. *R*_nat_ = 632 μg g^−1^ after 52 days, Table 1). Reduced Fe^2+^ was exclusively found in *R*_nat_ (Fig. 4) but not in *R*_cont_, where colloidal Fe was responsible for Fe mobilisation (Fig. 1b, Table 1). Although not thermodynamically favoured, some As(III) was released in R_cont_ (Fig. 3a) in the very beginning of reactor operation (until ~ 192 h, which corresponds to four exchanges of medium). The fact that As(III) was mobilised only initially at high rate as well as that during most reactor operation, pentavalent arsenic was found in *R*_cont_ only (Fig. 3) indicate the source of As(III) being desorption from the solid soil phase as a result of non-equilibrium conditions of liquid-saturated soil samples rather than being a result of As reduction. The amount of As(III) released (~ 45 μg g^−1^ = ~ 2% of total) is in coherence with a previous XAFS analysis of the initial soil that indicated that although As(III) is present mostly in the oxidised form, a minor fraction (~ 3%) may be present in the reduced form(Rajpert et al. 2016).

The mobilisation rates and the total amount of mobilised Mn were virtually identical in both setups (Fig. 1c), pointing towards Mn reductive dissolution prevailing throughout the reactor operation. Since redox conditions did not favour Fe or As reduction (Fig. 2), As was released either by colloidal Fe (Table 1) or as a result of reductive dissolution of Mn host phases. Both mobilisation mechanisms are of high importance regarding environmental risks. On the one hand, colloid-conferred element mobilisation may result in long distance transport of As (and other trace elements (Gomez-Gonzalez et al. 2018)). On the other hand, Mn-sequestered trace elements are easily mobilised because Mn phases are prone to reductive dissolution at moderate redox conditions (i.e. upon water logging induced by factors such as soil compaction and flooding). The second mechanism is in line with a previous study in which Mn phases, despite their much lower soil concentration, were correlated to mainly conferred As release (Rajpert et al. 2016).

Since Mn reduction occurred in both reactions, the higher overall amount of As released (~ 17 vs. ~ 9% of total) in *R*_nat_ was due to either Fe phases (i.e. colloidal mobilisation or reductive dissolution or both) or direct reduction of pentavalent As and desorption of As(III) from the solid phase. The higher share (Fig. 3) and overall amount of As(III) released (Table 1) confirmed the activity of autochthonous bacteria, either As resistant or dissimilatory As(V) reducing (see e.g. Stolz et al. 2006; Drewniak et al. 2010; Uhrynowski et al. 2017; Kruger et al. 2013). The existence and activity of an autochthonous dissimilatory As(V)-reducing bacterial community in the studied soil is in line with correlations observed in a previous study (Rajpert et al. 2016), where its proliferation resulted in a sudden arsenic release after As-bearing Fe and Mn pools were depleted by reduction.

Because of the considerable amount of As released already at moderately reducing conditions by Mn reductive dissolution, authors recommend implementing detailed monitoring systems and timely (bio)remediation in the Zloty Stok area. A calmative aspect of the study is the fact that in both reactors, induction of oxidising conditions resulted in immediate removal of As from the liquid phase (Fig. 1a) because of the sorption or co-precipitation of the trace element by precipitation of Fe/Mn phases (Fig. 1b, c).

In summary, the redox-stat reactor *R*_cont_ allowed suppression of both As and Fe reduction and shed more light on otherwise inconclusive mobilisation mechanisms (i.e. co-existing As(III) desorption, Mn(IV) reduction, As(V) reduction, colloidal Fe mobilisation and reductive Fe dissolution as occurring mechanisms in *R*_nat_). The technology described here is not limited to the study of As mobilisation and sequestration mechanisms, but can be expanded to study redox transformations of any major and trace elements (e.g. Fe, Mn, C, P, N, S, Cr, Cu, Co, As, Sb, Se, Hg, Tc, U). The detailed understanding of redox processes governing biogeochemical cycles of such (trace) elements is a key for both environmental risk analysis (as speciation determines bioavailability, toxicity and mobility) as well as exploitation of mechanisms in the frame of new (bio)remediation strategies. A few examples below underline the potential use of redox-stat bioreactors. Recently, a considerable seasonal variability of Sb leaching was found in outdoor lysimeters containing contaminated shooting range soils (Hockmann et al. 2015). Although under drained (i.e. high redox) conditions, high amount of Sb was mobilised (40 to 110 ppb in summer and winter, respectively), reducing conditions led to a fraction of Sb leaching (2–5 ppb), which was assigned to increased sorption of Sb(III) to Fe/Mn phases. With the proposed redox-stat CSTR, one may treat this soil forcing the redox conditions similar to those in drained lysimeters, preventing Sb(V) reduction to Sb(III), thus mobilising and ultimately removing Sb from contaminated soil.

Stimulating autochthonous dissimilatory metal-reducing microorganisms to remove hexavalent U(VI) from aquifers by uraninite-U(IV) precipitation has been suggested to prevent further downgradient spread of groundwater contamination (Anderson et al. 2003). However, the long-term reduction of U(VI) to U(IV) can be maintained only by suppression of autochthonous sulphate-reducing bacteria, since sulphide inhibits metal reducers. The application of the redox-stat reactor in actively preventing sulphate-reducing conditions might thus help to improve bioremediation of uranium-contaminated media.

Microbial bioremediation of selenium (Se) contamination usually exploits the conversion of water soluble, toxic Se oxyanions [Se(VI), Se(IV)] to water insoluble, elemental Se, which might be easily separated from the aqueous phase (Buchs et al. 2013). However, there is a risk of formation of selenide species at strongly reducing conditions. In metal-rich environments, selenide formation is of less concern (since metal selenides are extremely poorly water soluble) (Lenz et al. 2011). However, when metal as selenide scavengers are depleted, highly toxic H_2_Se may evolve from the aqueous phase. Therefore, a redox stat can be used to set *E*_h_ to values sufficiently low for allowing (Se) oxyanion reduction yet high enough to prevent formation of hydrogen selenide.

In summary, this study shed light on the role of the main host phases in arsenic mobilisation via suppression of underlying redox reactions. This and the few further examples provided (i.e. Sb, U and Se) underline the promising application of redox-stat systems in the study of biogeochemical cycles and in bioremediation of redox-sensitive trace elements.

## Electronic supplementary material

ESM 1 (PDF 97 kb)
