# Supplementary material for: Redox-stat bioreactors for elucidating mobilisation mechanisms of trace elements: an example of As-contaminated mining soils
Source: Appl Microbiol Biotechnol. 2018 Jun 21;102(17):7635–41. doi: 10.1007/s00253-018-9165-4 (PMC6097752; doi:10.1007/s00253-018-9165-4)
Supplement: Supplementary file 1 — (PDF 97 kb) [file 253_2018_9165_MOESM1_ESM.pdf]

Applied Microbiology and Biotechnology

SUPPLEMENTARY INFORMATION

"Redox-stat bioreactors for elucidating mobilisation mechanisms of trace elements: An example of As-contaminated mining soils"

Liwia Rajpert [1,2], Andreas Schäffer [2], Markus Lenz [1,3*]

[1] Institute for Ecopreneurship, School of Life Sciences, University of Applied Sciences and Arts Northwestern Switzerland, Gründenstrasse 40, 4132 Muttenz, Switzerland

[2] Institute for Environmental Research (Biology V), RWTH Aachen University, 52074 Aachen, Germany

[3] Sub-Department of Environmental Technology, Wageningen University, 6700 EV Wageningen, The Netherlands

[*]corresponding address: markus.lenz@fhnw.ch, (T) +41 612 285 686, (F) +41 614 674 290, ORCID ID orcid.org/0000-0001-6832-3218, Scopus ID 14031646900

Table S1. Elemental concentration (X-ray fluorescence) of intial Zloty Stok soil ($C_{initial}$) and soil upon termination of the reactor operation (C_{end}). Experimental recovery was calculated as the sum of C_{end} and cumulative element mobilized divided by $\mathbf{C_{Initial}}$ **× 100.**

Element	$C_{Initial}$ [µg/g]	C_{end} R_{nat} [µg/g]	Recovery R_{nat} [%]	C_{end} R_{cont} [µg/g]	Recovery R_{cont} [%]
**As**	2010±28	1625±17	97.8%	1727±40	95.1%
**Fe**	53867±1416	49437±257	94.5%	51427±248	96.6%
**Mn**	1164±75	548±9	101.8%	606±8	106.0%

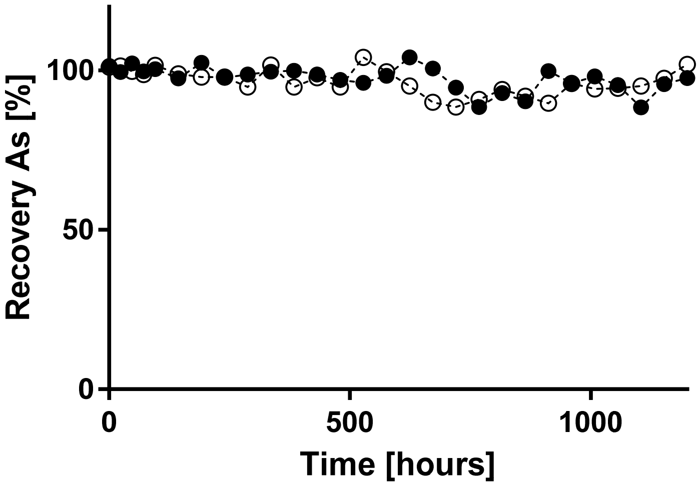

Fig. S1. Experimental recovery of arsenic species as [(As(III) + As(V)) / As total] × 100 during reactor operation before re-oxidation. Average recovery R_{cont} = 96.6 ± 4.1 %; Average recovery R_{nat} = 97.7 ± 3.9 %.
